# Association of Short-Form Video Use With Attention Control and Sleep Quality Among Medical Students: A Cross-Sectional Study

**DOI:** 10.7759/cureus.108156

**Published:** 2026-05-02

**Authors:** Bilal Asad Mohammed, Snehika Allampally, Pulluri Sadanandam, Megha Jharbade, Shilpa Samayam

**Affiliations:** 1 Community Medicine, Government Medical College, Siddipet, Siddipet, IND; 2 Community Medicine, Government Medical College, Maheshwaram, Mangalpally, IND

**Keywords:** attention control, digital hygiene, short-form videos, sleep quality, social media use

## Abstract

Background: The growing popularity of short-form videos (SFVs) on platforms like Instagram (Meta Platforms, Inc., Menlo Park, California, USA) and TikTok (ByteDance Ltd, Beijing, China) has raised concerns regarding their potential association with attention and sleep. Due to their fast-paced and highly stimulating characteristics, SFVs may influence attentional processes and sleep patterns. This study aimed to assess the association between short-form video use, attention control and sleep quality among medical students.

Objectives: To determine the extent of short-form video usage and examine the association of usage frequency and viewing duration with attention control and sleep quality among medical students.

Methods: A cross-sectional study was conducted from January to March 2025 among 342 medical students using convenience sampling. Data were collected using a pre-designed, self-administered questionnaire assessing sociodemographic characteristics, short-form video (SFV) usage patterns, attention control (using the Attention Control Scale), and sleep quality (using the Pittsburgh Sleep Quality Index). Descriptive statistics and chi-square tests were applied to assess associations.

Results: Among the 342 medical students, 268 (78.4%) reported watching short-form videos daily, with 179 (52.3%) reporting more than one hour of daily use. A significant association was observed between higher frequency of app opening and lower attention control (χ²(6) = 43.955, p < 0.001, Cramer’s V = 0.25). Viewing duration was not significantly associated with attention control (χ²(6) = 6.036, p = 0.419, Cramer’s V = 0.09). Longer daily viewing duration was significantly associated with poor sleep quality (χ²(3) = 8.403, p = 0.038, Cramer’s V = 0.16), whereas frequency of app opening was not significantly associated with sleep quality (χ²(3) = 7.645, p = 0.054, Cramer’s V = 0.15).

Conclusion: Increased duration of short-form video viewing was associated with poorer sleep quality, while higher frequency of app opening was associated with lower attention control among medical students. These findings suggest possible associations between short-form video use with attention control and sleep quality, highlighting the need for further longitudinal studies to better understand these associations and inform strategies promoting digital hygiene and responsible media use.

## Introduction

In recent years, short-form video (SFV) platforms like TikTok (ByteDance Ltd, Beijing, China), Instagram (Meta Platforms, Inc., Menlo Park, California, USA) Reels, and YouTube (Google LLC, Mountain View, California, USA) Shorts have become increasingly popular, especially among adolescents and young adults [1]. Short-form videos (SFVs) are brief, user-generated social media videos typically ranging from a few seconds to several minutes in duration [2]. Short-form video platforms deliver rapidly changing, algorithm-driven content designed to maximize user engagement through brief, highly stimulating exposures [1,3]. While these features make the platforms entertaining and creatively stimulating, there is growing concern about their possible association with attention control and sleep quality, two crucial aspects of cognitive and emotional health [4-6].

In particular, short-form video (SFV) usage may also be associated with sleep quality, as increased exposure to screen-based content especially during nighttime has been linked to delayed sleep onset and reduced overall sleep quality [7-10]. Students, as frequent users of social media, may be particularly vulnerable to these associations. SFVs are designed for quick consumption, encouraging frequent attention switching and limiting sustained focus. Several studies suggest that this constant switching may be associated with reduced attention control and executive functioning [11-13]. Montag et al. highlighted how digital media designs can reinforce fragmented attention cycles and reduce cognitive endurance [3], while Elhai et al. demonstrated that problematic social media use is associated with challenges in executive functioning and emotional regulation [12].

Sleep quality is another major concern. Nighttime screen use, particularly on personal devices, has been widely shown to disrupt healthy sleep routines. Exposure to light from electronic screens may reduce melatonin secretion and disrupt circadian rhythm regulation, potentially delaying sleep onset and impairing sleep quality [7-9]. Chang et al. found that using light-emitting devices before bed not only reduces sleep quality but also affects next-day alertness and performance [10]. Similarly, Exelmans and Van den Bulck found that bedtime phone use is associated with increased sleep latency and daytime fatigue [8,9]. Another relevant behavior is 'bedtime procrastination', where individuals knowingly delay sleep to keep consuming digital media despite being tired. This has been strongly linked to lower self-control and compulsive smartphone use, both of which are often observed among regular SFV users [14-17].

Early studies suggest that excessive SFV use may be associated with psychological issues like social anxiety, academic procrastination, and impaired cognitive control [11-13,18-20]. However, the evidence remains limited. This cross-sectional study therefore aims to examine the association of short-form video use with attention control and sleep quality among medical students. By examining this emerging digital media format, the study provides data that may support the development of interventions aimed at improving digital well-being.

## Materials and methods

A cross-sectional study was conducted among medical students of Government Medical College, Siddipet, Telangana, from January to March 2025. The objective was to examine the association of short-form video (SFV) use with attention control and sleep quality. The study was carried out after obtaining approval from the Institutional Human Ethics Committee of Government Medical College, Siddipet, Telangana, India (Approval No. GMCS/IECBHR2025/154; dated: 01-03-2025).

The study population comprised students from the first year to the final year enrolled during the study period. A total of 342 eligible students who provided informed consent were included using a convenience sampling approach. A formal sample size calculation was not performed, and the sample size was based on feasibility during the study period. The Google Forms (Google LLC, Mountain View, California, USA) survey link, which contained study information, consent, and the questionnaire, were shared via online student groups and during classroom visits, where a QR code was provided after explaining the study. Inclusion criteria involved students who reported using short-form video platforms and provided informed consent. Students with self-reported conditions known to significantly affect attention or sleep (e.g., attention-deficit/hyperactivity disorder (ADHD), anxiety disorders) were excluded to minimize potential confounding. Students who declined participation or did not complete the questionnaire were also excluded from the final analysis.

Data were collected using a pre-designed, self-administered questionnaire distributed via Google Forms. The non-validated portions of the questionnaire were not formally pilot tested prior to administration. The tool included sections on demographic details, patterns of short-form video usage, and validated scales (Pittsburgh Sleep Quality Index (PSQI) [21] and Attention Control Scale (ACS) [22]) to assess outcomes. Patterns of short-form video usage were assessed using questions on average daily viewing duration (<30 min, 30-60 min, 61-180 min, >180 min) and frequency of app usage per day (<5 times, 5-10 times, 11-20 times, >20 times). All data, including short-form video usage patterns, were self-reported by participants.

Sleep quality was measured using the Pittsburgh Sleep Quality Index (PSQI), a validated instrument with a global score ranging from 0 to 21 [21]. A score >5 was considered indicative of poor sleep quality, while a score ≤5 indicated good sleep quality, and participants were categorized accordingly. Attention control was assessed using the Attention Control Scale (ACS), a 20-item validated self-report measure developed by Derryberry and Reed (2002) [22]. Each item is rated on a four-point Likert scale, yielding a total score ranging from 20 to 80, with higher scores indicating better attentional control. As no standardized categorical cutoffs for ACS scores have been established, scores were divided into low (20-40), moderate (41-60), and high (61-80) groups using equal-interval division of the total score range for analytical purposes.

Descriptive statistics such as frequencies, percentages, means, and standard deviations were calculated. The chi-square test was applied to evaluate associations between short-form video usage and outcome variables. Additional exploratory analyses were performed to assess (1) the association between sleep quality and attention control, to determine whether these variables were interrelated and could potentially influence the study outcomes, and (2) gender-based differences in attention control and sleep quality. These analyses were exploratory in nature and were not part of the primary study objective. Effect size for chi-square tests was assessed using Cramer’s V. A p-value of less than 0.05 was considered statistically significant. Data were analyzed using Microsoft Excel (Microsoft Corporation, Redmond, Washington, USA), Epi Info version 7.1 (Centers for Disease Control and Prevention (CDC), Atlanta, Georgia, USA), and SPSS version 26 (trial version, IBM Corp, Armonk, New York, USA). Confidentiality of participant information was strictly maintained throughout the study, and informed consent was obtained from all participants prior to inclusion.

## Results

Sociodemographic profile

The present study included 342 students from the Government Medical College, Siddipet. Of the total participants, 127 (37.1%) were males and 215 (62.9%) were females, indicating that approximately two-thirds of the study population were female. A majority of the respondents (88.6%) identified as Hindu, followed by Muslim (6.1%), Christian (4.4%), and other religions (0.9%). Regarding socioeconomic status, most participants (63.2%) belonged to the middle class, followed by 19% in the upper-middle class and 14.3% in the lower-middle class. Only a small proportion belonged to the upper-class (2.3%) and lower-class (1.2%).

Patterns of short-form video usage

**Figure 1:**
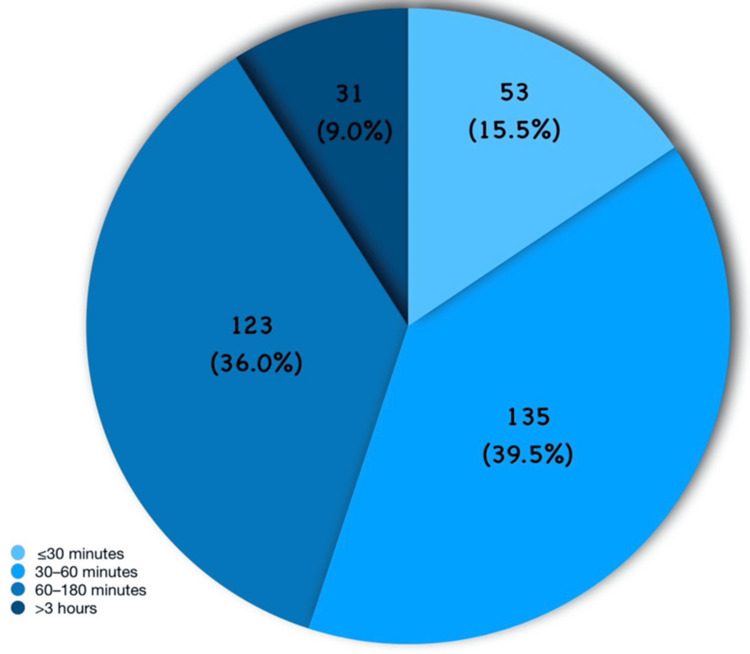
Distribution of study population (N = 342) according to daily duration of short-form video usage

Regarding time spent on short-form videos, 53 (15.5%) participants reported watching for <30 minutes per day, 135 (39.5%) for 30-60 minutes, and 123 (36.0%) for 61-180 minutes. Only 31 (9.1%) participants reported viewing content for more than three hours per day. As shown in Figure 1, the majority of participants reported watching short-form videos for 30-60 minutes per day.

**Figure 2:**
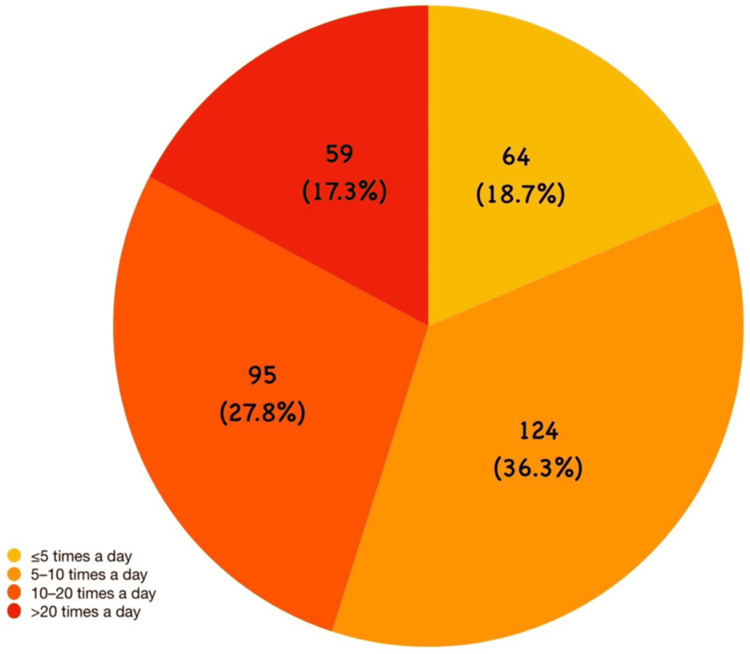
Distribution of study population (N=342) according to daily frequency of short-form video application usage

The distribution of app-opening frequency showed that 124 (36.3%) participants reported opening the application 5-10 times daily, 95 (27.8%) reported 11-20 openings per day, 64 (18.7%) reported <5 openings per day, and 59 (17.3%) reported >20 openings per day. Figure 2 illustrates that the largest proportion of participants opened the application 5-10 times per day.

Association between short-form video usage and sleep quality

A significant association was observed between sleep quality and daily screen time spent on short-form videos (χ²(3) = 8.403, p = 0.038), with a weak effect size (Cramer’s V = 0.16). Participants with good sleep were more likely to report shorter viewing durations (<30 minutes: 21.1% vs 9.9%), whereas participants with poor sleep showed relatively higher proportions in longer viewing categories, particularly 61-180 minutes (39.8% vs 32.2%). Overall, the distribution suggests a shift toward longer viewing durations among poor sleepers, as shown in Table 1.

**Table 1:** Association between short-form video usage and sleep quality among study population Chi-square test was used to assess the association (χ²(3) = 8.403, p = 0.038; Cramer’s V = 0.16). n: number of participants; %: row-wise percentages.

Quality of Sleep	Duration of Watch Time (per day)				Total
	<30 minutes	30 minutes to 60 minutes	61 minutes to 180 minutes	More than 3 hours	
Poor	17 (9.9%)	70 (40.9%)	68 (39.8%)	16 (9.4%)	171 (100%)
Good	36 (21.1%)	65 (38%)	55 (32.2%)	15 (8.8%)	171 (100%)
Total	53 (15.5%)	135 (39.5%)	123 (36%)	31 (9.1%)	342 (100%)

Although the association between sleep quality and frequency of app opening was not statistically significant (χ²(3) = 7.645, p = 0.054), with a weak effect size (Cramer’s V = 0.15), a marginal difference in distribution was observed, as shown in Table 2, with participants reporting poor sleep exhibiting slightly higher proportions in the highest usage category (>20 openings per day; 18.7% vs 15.8%).

**Table 2:** Association of study population between quality of sleep and frequency of app opening Chi-square test was used to assess the association (χ²(3) = 7.645, p = 0.054; Cramer’s V = 0.15). n: number of participants; %: row-wise percentages.

Quality of Sleep	Frequency of App Opening				Total
	<5 times	5 to 10 times	11 to 20 times	More than 20 times	
Poor	23 (13.5%)	70 (40.9%)	46 (26.9%)	32 (18.7%)	171 (100%)
Good	41 (24.0%)	54 (31.6%)	49 (28.7%)	27 (15.8%)	171 (100%)
Total	64 (18.7%)	124 (36.3%)	95 (27.8%)	59 (17.3%)	342 (100%)

Association between short-form video usage and attention control

Frequency of App Opening and Attention Control

A statistically significant association was observed between attention control and the frequency of app opening (χ²(6) = 43.955, p < 0.001), with a moderate effect size (Cramer’s V = 0.25). As presented in Table 3, participants with high attention control predominantly reported lower-frequency app use (<5 openings/day; n = 8, 66.7%), whereas those with low attention control were disproportionately represented in the highest usage category (>20 openings/day; n = 10, 58.8%). This distribution suggests a notable association between attention control and app-opening frequency.

**Table 3:** Association of study population between attention control and frequency of app opening Chi-square test was used to assess the association (χ²(6) = 43.955, p < 0.001; Cramer’s V = 0.25). n: number of participants; %: row-wise percentages.

Attention Control	Frequency of App Opening				Total
	<5 times	5 to 10 times	11 to 20 times	More than 20 times	
High	8 (66.7%)	3 (25%)	0 (0%)	1 (8.3%)	12 (100%)
Moderate	55 (17.6%)	115 (36.7%)	95 (30.4%)	48 (15.3%)	313 (100%)
Low	1 (5.9%)	6 (35.3%)	0 (0%)	10 (58.8%)	17 (100%)
Total	64 (18.7%)	124 (36.3%)	95 (27.8%)	59 (17.3%)	342 (100%)

Duration of Viewing and Attention Control

In an additional analysis, no significant association was found between attention control and time spent watching short-form videos per day (χ²(6) = 6.036 , p = 0.419), with a weak effect size (Cramer’s V = 0.09), indicating that attention control levels were similarly distributed across viewing duration categories within this sample.

Association between sleep quality and attention control

Distribution of Attention Control and Sleep Quality

Among the participants, 91.5% demonstrated moderate attention control, 5% exhibited low, and 3.5% exhibited high attention control. Sleep quality was evenly distributed in the study population, with equal proportions (50%) reporting good and poor sleep.

Association of Study Population Between Quality of Sleep and Attention Control

No significant association was found between sleep quality and attention control (χ²(2) = 4.244, p = 0.120), with a weak effect size (Cramer’s V = 0.11). In both sleep quality groups, the majority of participants demonstrated moderate attention control (90.6% among poor sleepers vs 92.4% among good sleepers), while the proportions exhibiting high attention control were low in both groups (2.3% and 4.7%, respectively). As presented in Table 4, the distribution of attention control levels was similar across sleep quality categories, suggesting minimal variation in attention control based on sleep quality within this sample.

**Table 4:** Association of study population between quality of sleep and attention control Chi-square test was used to assess the association (χ²(2) = 4.244, p = 0.120; Cramer’s V = 0.11). n: number of participants; %: row-wise percentages.

Quality of Sleep	Attention Control			
	High n (%)	Low n (%)	Moderate n (%)	Total
Poor	4 (2.3%)	12 (7%)	155 (90.6%)	171 (100%)
Good	8 (4.7%)	5 (2.9%)	158 (92.4%)	171 (100%)
Total	12 (3.5%)	17 (5%)	313 (91.5%)	342 (100%)

Gender-based associations

Exploratory analyses were conducted to examine whether gender influenced cognitive or sleep-related outcomes. No statistically significant association was noted between gender and attention control (χ²(2) = 2.597 , p = 0.273), with a weak effect size (Cramer’s V = 0.09). Similarly, no statistically significant association was observed between gender and sleep quality (χ²(1) = 0.113, p = 0.737), with a weak effect size (Cramer’s V = 0.02). The distribution of good and poor sleep quality was nearly identical between male and female participants.

## Discussion

The present study examined the association of short-form video (SFV) usage with attention control and sleep quality among medical students. Although viewing duration was not significantly associated with attention control, higher frequency of SFV use was associated with lower attention control, suggesting that patterns of engagement may play a more relevant role than total exposure time. Notably, exploratory analyses were conducted to assess relationships beyond the primary study objectives. No significant association was observed between sleep quality and attention control. Gender was also not significantly associated with either attention control or sleep quality, suggesting that the primary findings were not significantly associated with these factors.

Association with attention control

A statistically significant association was observed between higher frequency of short-form video use and lower attention control scores. Notably, this association demonstrated a moderate effect size, suggesting that frequency of engagement may be a more relevant behavioral indicator than viewing duration. This finding is consistent with previous studies indicating that engagement with rapidly shifting digital content and simultaneous media exposure is associated with alterations in working memory performance and attentional control. Uncapher et al. demonstrated that individuals frequently engaged in media multitasking showed weaker performance in both working memory and long-term memory tasks [11]. Similarly, Wilmer et al. reported that habitual smartphone use, particularly for entertainment, was associated with reduced cognitive control and executive functioning [13].

Montag et al. emphasized that short-form video platforms, by delivering highly stimulating and rapidly changing content, may reinforce fragmented attention cycles and reduce cognitive endurance [3]. In this context, continuous exposure to short, engaging videos may reinforce a preference for frequent novelty and rapid reward processing, thereby reducing tolerance for sustained focus, which is essential in academic and clinical environments. In contrast, viewing duration was not significantly associated with attention control in this study, suggesting that frequency of engagement rather than total time spent may be more relevant in relation to attentional processes.

Association with sleep quality

This study identified a statistically significant association between longer duration of short-form video usage and poor sleep quality. Participants reporting extended daily viewing were more likely to report poor sleep quality. These findings are consistent with prior research suggesting that increased screen exposure, particularly during evening hours, may be associated with delayed sleep onset and reduced sleep quality. Cain and Gradisar reviewed multiple studies showing that electronic media use in adolescents interferes with melatonin production, sleep duration, and sleep efficiency [7]. Chang et al. further reported that evening exposure to light-emitting devices negatively impacts circadian timing and next-morning alertness [10]. Additionally, a phenomenon termed ‘bedtime procrastination' refers to the intentional postponement of sleep despite the absence of external constraints, often driven by prolonged digital engagement. Kroese et al. described bedtime procrastination as a self-regulatory failure, often exacerbated by highly engaging content such as SFVs, which promote continuous use through autoplay and algorithmic feeds [15]. In contrast, frequency of app opening was not significantly associated with sleep quality, suggesting that total screen exposure time may be more relevant to sleep-related outcomes in this sample.

Broader psychosocial and academic implications

Beyond attention and sleep, prior research suggests that SFV overuse may be associated with broader psychological concerns, including emotional dysregulation and academic procrastination. Elhai et al. found that SFV addiction was significantly linked with higher levels of depression, anxiety, and stress among university students [12]. Similarly, Xie et al. observed that SFV use contributed to academic procrastination, mediated by poor emotion regulation and low self-control [5]. While our study did not directly measure psychological outcomes or academic performance, the reported fatigue, poor concentration, and sleep disturbances among SFV users may act as early indicators of these adverse outcomes.

Limitations

Being cross-sectional in design, causal relationships cannot be established. A formal sample size calculation was not performed, which may have affected the statistical power of the study. Self-reported measures of short-form video use, attention control, and sleep quality are subject to recall and social desirability bias. App opening frequency was used as a proxy measure of exposure, which may not fully reflect actual engagement patterns. Attention control was categorized using equal-interval ranges due to the absence of established cutoffs; therefore, these categories should be interpreted with caution. Objective measures of sleep and attention were not employed, and potential confounding variables were not adjusted for as multivariate analysis was not performed. The use of convenience sampling may have introduced selection bias, and the single-institution setting may limit the external validity and generalizability of the findings.

## Conclusions

Increased duration of short-form video use was associated with poorer sleep quality, while higher frequency of app opening was associated with lower attention control among medical students. These findings suggest possible associations between short-form video use, attention control, and sleep quality. However, given the cross-sectional design and lack of adjustment for potential confounders, these findings should be interpreted with caution. Promoting digital hygiene, including limiting screen time and reducing frequent app-checking behavior, may be beneficial. Further longitudinal studies are needed to better understand these relationships.
